# Prediction of cognitive impairment in Parkinson’s disease using TabPFN: an integrative analysis of clinical data, longitudinal trajectories, and lipidomics

**DOI:** 10.3389/fnagi.2026.1905374

**Published:** 2026-09-30

**Authors:** Zikai Zhou, Jie Liu, Lijuan Wang, Hong Lv, Wencan Jiang, Ling Huang, Guojun Zhang, Ruimin Ma

**Affiliations:** 1 Laboratory Diagnosis Center, Beijing Tiantan Hospital, Capital Medical University, Beijing, China; 2 Laboratory Diagnosis Platform for Nervous System Infectious Diseases, Laboratory for Clinical Medicine, Capital Medical University, Beijing, China; 3 Beijing Key Laboratory of Biomarker Screening and Translational Research in Neurological Diseases, Beijing, China

**Keywords:** cognitive impairment, longitudinal analysis, machine learning, Parkinson’s disease, triglycerides

## Abstract

**Background:**

Cognitive impairment is a common and disabling non-motor manifestation of Parkinson’s disease (PD). Early identification of patients at high risk remains challenging, and the metabolic mechanisms underlying cognitive decline are not fully understood.

**Methods:**

We conducted a retrospective cohort study including PD patients who were cognitively normal at baseline. A machine learning model based on a Tabular Prior-data Fitted Network (TabPFN) was developed using routinely collected clinical and laboratory data to predict future cognitive impairment. Model performance was evaluated using stratified group cross-validation and external validation in the Parkinson’s Progression Markers Initiative (PPMI) cohort. Model interpretability was assessed using SHapley Additive exPlanations (SHAP). Longitudinal analyses and untargeted lipidomics were performed to explore temporal and molecular alterations associated with cognitive decline.

**Results:**

Among 91 patients in the internal cohort, 45 developed cognitive impairment during follow-up. The TabPFN model achieved an AUC of 0.822 (95% CI 0.709–0.889) in internal validation and 0.742 (95% CI 0.695–0.783) in external validation. In internal benchmark comparisons, TabPFN showed numerically higher discrimination than logistic regression (AUC = 0.728) and random forest (AUC = 0.787). MoCA and age were the most influential predictors, while triglycerides (TG) also contributed to model predictions. In the longitudinal analysis, TG trajectories differed between converters and stable participants after adjustment for relevant covariates (group-by-time interaction: β = −0.146, 95% CI − 0.248 to −0.044; *p* = 0.005). In the independent lipidomics cohort, although total TG abundance did not differ significantly between cognitive groups, several individual lipid species, including specific TG species, showed differential abundance.

**Conclusion:**

A TabPFN-based model using routinely available clinical and biochemical variables showed promising discrimination for future cognitive impairment in PD. TG contributed to prediction, and longitudinal analyses identified different TG trajectories between converters and stable participants. The cross-sectional lipidomics findings provide complementary, hypothesis-generating information about molecular lipid heterogeneity but do not establish the mechanism underlying the longitudinal TG pattern. Prospective multicenter validation is required before clinical implementation.

## 1 Introduction

Parkinson’s disease (PD) is the second most common neurodegenerative disease, characterized by motor symptoms (bradykinesia, rigidity, and resting tremor) as well as non-motor symptoms (Postuma et al., 2018). Cognitive impairment is the most common non-motor symptom in PD, and most patients with PD will have cognitive dysfunction in the course of their disease (Safarpour and Willis, 2016). Cognitive impairment is a significant contributor to disability in PD patients that can compromise their quality of life and independence, imposing a substantial economic burden (Guo et al., 2019; Rektorova, 2019; Vossius et al., 2011). Despite the clinical urgency, early detection of those at high risk for cognitive decline remains challenging. Current “gold standard” biomarkers, such as cerebrospinal fluid (CSF) assays and positron emission tomography (PET) imaging, provide valuable insights into underlying neuropathology (Bidesi et al., 2021; Li et al., 2025; Parnetti et al., 2019). However, their clinical utility is limited by invasiveness, high cost, and restricted accessibility, which hinder their widespread implementation in routine practice, necessitating the development of accurate, non-invasive, and cost-effective predictive tools.

Emerging evidence suggests that lipid metabolism alterations may play a critical role in the pathophysiology of PD (Pennetta and Welte, 2018). Cross-sectional studies have associated elevated serum triglyceride levels with PD-related mild cognitive impairment, while lipidomic analyses have revealed abnormalities in triglycerides, sphingolipids, phospholipids, and other lipid pathways (Galper et al., 2022; Huang et al., 2018). However, these predominantly cross-sectional findings do not establish whether routinely measured triglyceride levels contribute to the prospective prediction of cognitive impairment or how triglyceride levels change within individuals as cognitive impairment develops. The temporal relationship between lipid metabolism and cognitive progression in PD therefore remains incompletely characterized.

Machine learning (ML) has emerged as a powerful tool for modeling complex, high-dimensional clinical data and holds promise for improving risk stratification in neurodegenerative diseases. Previous PD cognitive prediction studies using ML have used demographic and clinical variables together with cognitive assessments, cerebrospinal fluid biomarkers, genetic data, and neuroimaging to predict incident cognitive impairment, dementia conversion, or longitudinal changes in cognitive scores (Caspell-Garcia et al., 2017; Luo et al., 2025). These studies demonstrate the feasibility of predicting cognitive outcomes in PD but frequently involve specialized multimodal measurements, difficult-to-obtain biological samples, fixed outcome assessment time points, or longitudinal modeling architectures that require multiple prior observations. The emergence of the Tabular Prior-data Fitted Network (TabPFN) offers a promising avenue to address this challenge (Hollmann et al., 2025). By leveraging in-context learning to approximate Bayesian inference, TabPFN is uniquely suited for clinical tabular data, offering robust generalization even with limited samples and complex missingness. In the present study, we evaluated whether TabPFN could estimate future cognitive impairment risk using routinely available clinical and laboratory variables.

Accordingly, this study had two related but distinct objectives. The primary objective was predictive: to develop and externally evaluate a TabPFN-based model using routinely collected clinical and biochemical data to estimate the probability of cognitive impairment over a specified future prediction window in patients with PD. The secondary objective was exploratory and focused on patterns related to triglycerides (TG). Longitudinal clinical records were analyzed to characterize within-patient changes in routine TG levels, while an independently recruited cross-sectional lipidomics cohort was used to examine lipid-class and lipid-species differences associated with cognitive status.

## 2 Methods

### 2.1 Study population

This was a retrospective cohort study. The internal cohort was retrospectively identified from patients admitted to the Department of Neurology at Beijing Tiantan Hospital between October 2018 and September 2025. The study protocol was approved by the Institutional Review Board (IRB) of Beijing Tiantan Hospital, Capital Medical University (approval no. [KY2025-271-02]), with all data strictly anonymized.

#### 2.1.1 Inclusion criteria

Patients were eligible for inclusion if they met the following criteria:

1. A clinical diagnosis of primary Parkinson’s disease (PD) based on the 2015 Movement Disorder Society (MDS) clinical diagnostic criteria;
2. Presence of at least two inpatient admissions, with at least two comprehensive records separated by an interval of at least three months to ensure adequate temporal separation between assessments;
3. A baseline cognitive status of “No Cognitive Impairment” (NCI) at the first eligible admission, determined according to established MDS criteria based on cognitive assessments and clinical evaluation.

#### 2.1.2 Exclusion criteria

To ensure cohort homogeneity and the reliability of cognitive outcomes, we excluded patients based on:

1. Presence of atypical parkinsonism or secondary parkinsonian syndromes (e.g., multiple system atrophy, progressive supranuclear palsy, or vascular parkinsonism);
2. Acute conditions precluding reliable cognitive assessment, such as delirium, severe systemic infection or coma;
3. Current or recent use of anticholinergic medications, which are known to have significant confounding effects on cognitive performance (Campbell et al., 2009).
4. Current use of any lipid-lowering medication.
5. Age younger than 18 years.

Eligible longitudinal medical records for each patient were ordered chronologically. Records obtained after the first inpatient record documenting cognitive impairment, defined as either mild cognitive impairment (MCI) or Parkinson’s disease dementia (PDD), were not included.

For construction of the primary prediction dataset, only one visit pair (T, T1) was retained for each patient. T was defined as the first eligible visit at which the patient had no cognitive impairment (NCI). For patients who subsequently developed cognitive impairment, T1 was defined as the first visit documenting MCI or PDD. For patients who remained cognitively unimpaired throughout follow-up, T1 was defined as the last eligible visit. Intermediate visits were not used to construct additional prediction pairs. Thus, each patient contributed one independent observation to the primary prediction analysis, and all clinical predictor values were obtained at T, when the patient was cognitively unimpaired. The interval between T and T1 was included as the prediction horizon.

Intermediate eligible records were retained for the longitudinal TG analysis. Consequently, patients with more than two eligible records could contribute multiple TG measurements to that analysis, and within-patient correlation was accounted for using a linear mixed-effects model.

External validation was performed using data from the Parkinson’s Progression Markers Initiative (PPMI). Eligibility criteria were aligned with those of the internal cohort where applicable. PPMI participants were required to belong to the PD cohort, have at least two eligible cognitive assessments, have no cognitive impairment at the first eligible assessment, and satisfy the applicable medication and follow-up-interval criteria. The PPMI cohort was used exclusively for external validation and was not involved in model training.

Participant selection for the internal prediction cohort, longitudinal TG analysis, PPMI external-validation cohort, and independent prospective lipidomics cohort is summarized in Figure 1.

**Figure 1:**
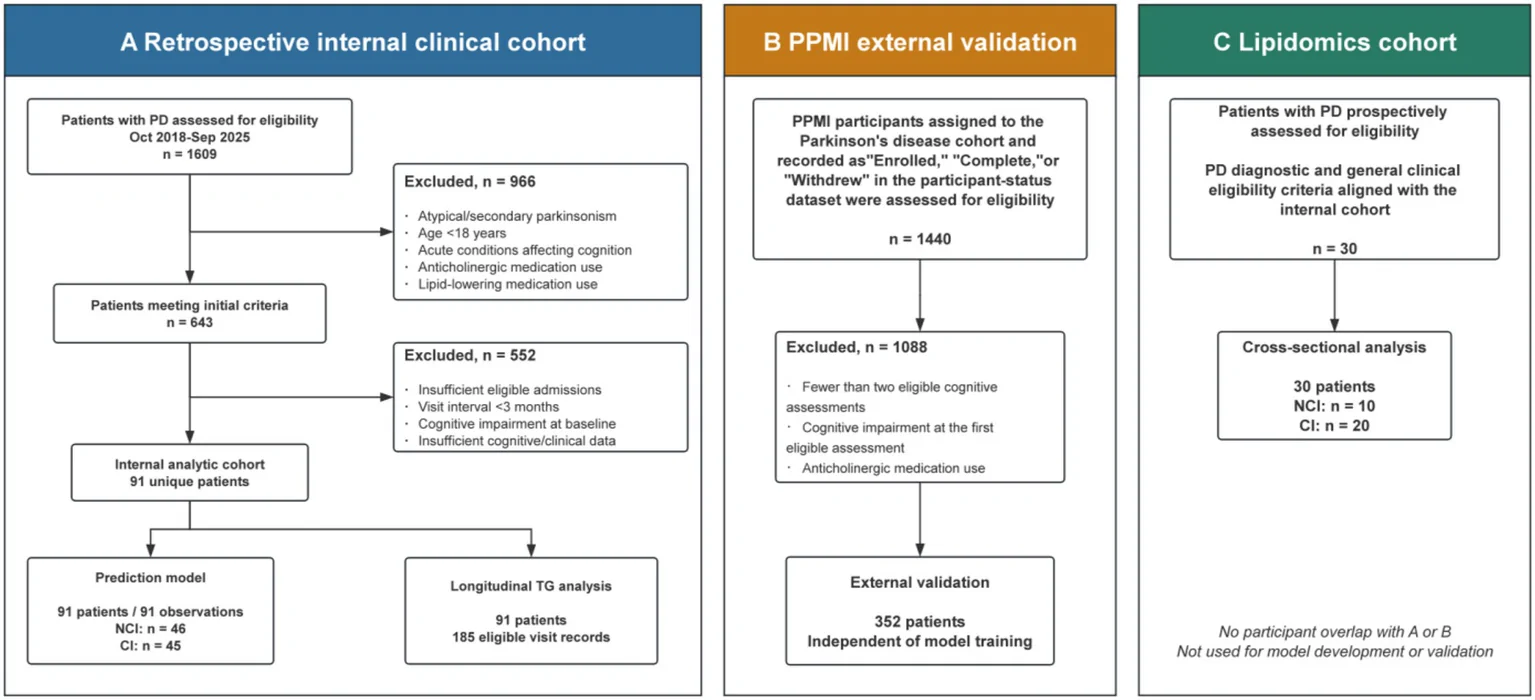
Participant flow and construction of the three analytic cohorts. **(A)** The retrospective internal clinical cohort was derived from patients with Parkinson’s disease (PD) admitted between October 2018 and September 2025. After application of the eligibility criteria, 91 patients were included in the prediction analysis, comprising 46 patients who remained cognitively unimpaired (NCI) and 45 who developed cognitive impairment (CI). Repeated eligible records from these 91 patients were also included in the longitudinal triglyceride (TG) analysis. **(B)** The Parkinson’s Progression Markers Initiative (PPMI) cohort was used exclusively for independent external validation and was not involved in model training. **(C)** The independently and prospectively recruited lipidomics cohort comprised 30 patients with PD, including 10 with NCI and 20 with CI. This cohort was used only for cross-sectional lipidomic analysis and had no participant overlap with either the internal clinical cohort or the PPMI cohort. The lipidomics cohort was not used for model development or validation. CI, cognitive impairment; NCI, no cognitive impairment; PD, Parkinson’s disease; PPMI, Parkinson’s Progression Markers Initiative; TG, triglyceride.

### 2.2 Outcome definition

The primary outcome was the development of cognitive impairment, defined as the first occurrence of either mild cognitive impairment (PD-MCI) or Parkinson’s disease dementia (PDD) during follow-up. These conditions were pooled because the primary clinical objective was to identify patients transitioning from a cognitively unimpaired state to any clinically relevant cognitive impairment. Moreover, a first recorded diagnosis of PDD may also have been preceded by an unobserved MCI stage. Nevertheless, because PD-MCI and PDD represent clinically distinct levels of cognitive impairment, component-specific sensitivity analyses were performed separately for first-recorded PD-MCI and PDD.

In the internal cohort, cognitive outcomes were determined according to the Movement Disorder Society (MDS) criteria, including the MDS Task Force criteria for PD-MCI and the MDS clinical diagnostic criteria for PDD (Litvan et al., 2012; Postuma et al., 2015). Diagnoses were based on a combination of standardized cognitive assessments (Montreal Cognitive Assessment, MoCA) and clinical evaluation by experienced neurologists. In the external PPMI cohort, cognitive status was defined based on the diagnostic labels provided in the PPMI database. These classifications are derived from standardized neuropsychological assessments and consensus diagnostic criteria implemented within the PPMI study protocol.

The event time was defined as the date of the first inpatient record documenting cognitive impairment. For patients who did not develop cognitive impairment, data were censored at the last available clinical encounter.

### 2.3 Predictor variables

Candidate predictors were derived from routinely collected clinical and laboratory data at each inpatient visit. These included demographic characteristics (e.g., age, sex, and education level), disease-related variables (e.g., disease duration and motor severity assessed by UPDRS-III), non-motor symptoms, cognitive performance, and laboratory biomarkers (lipid profile, inflammatory markers, and metabolic indicators). Serum triglyceride and other biochemical measurements were obtained from blood samples collected under fasting conditions as part of routine inpatient laboratory testing. In addition, a predefined prediction window (in months) was included as a model input to enable time-dependent risk estimation of future cognitive impairment. The prediction horizon was defined as the time, in months, from the index assessment to the assessment at which the cognitive outcome was observed. During model development, each observation consisted of an index visit (T), at which the patient was cognitively unimpaired, and the next eligible assessment (T1), the elapsed time between T and T1 is the prediction horizon. For the intended clinical application, it represents a user-specified future interval: the model takes the patient’s clinical characteristics at the current visit together with the requested prediction horizon as inputs and returns the corresponding predicted probability of CI. Predictions at different horizons can therefore be generated while holding the current clinical characteristics fixed. Missing data were handled natively by the TabPFN model without imputation. For external validation, unavailable features were retained and encoded as missing values.

### 2.4 Feature selection

Predictor selection was performed using a hybrid strategy combining clinical knowledge and data-driven methods. A set of clinically relevant variables was pre-specified and retained in all models, including age at visit, motor severity (UPDRS-III), baseline cognitive performance (MoCA), and the predefined prediction window (in months), based on prior literature and clinical relevance. To explore additional predictive features, a Random Forest model was used to estimate feature importance. Candidate variables were ranked according to their importance scores, and the top-ranked features were considered as exploratory predictors. To prevent information leakage, feature selection procedures were conducted within each cross-validation fold.

### 2.5 Model development

Model development was performed using the Tabular Prior-data Fitted Network (TabPFN), a pretrained transformer-based foundation model designed for tabular data and optimized for small-sample learning, it pretrained on a large collection of synthetically generated datasets representing diverse causal structures, feature distributions, and missing-data patterns (Hollmann et al., 2025), so missing values can be retained as NaN and passed directly to TabPFN. During application, the labelled training observations within each cross-validation fold were supplied as context, and predictions for the held-out observations were generated through in-context learning without gradient-based model fitting on the study dataset.

A stratified group 5-fold cross-validation framework was adopted to ensure robust model evaluation while preventing information leakage. Patient ID was used as the grouping variable to ensure that all records from the same individual were assigned exclusively to either the training or testing set within each fold. Within each fold, the model was trained on the training subset and evaluated on the held-out test subset. For the primary analysis, a binary classification framework was used, in which mild cognitive impairment (MCI) and Parkinson’s disease dementia (PDD) were combined into a single outcome category defined as cognitive impairment (CI).

The model generated predicted probabilities for the target outcome, which were used for downstream performance evaluation. The TabPFN model was applied using default hyperparameters without additional tuning. The trained model can be applied to new individuals by inputting the same set of predictors into the TabPFN framework, which outputs predicted probabilities of cognitive impairment.

Model development and validation were implemented in Python version 3.13.7 using the TabPFN v2.5.

### 2.6 Validation strategy

Model performance was evaluated using both internal and external validation. Internal validation was conducted using stratified group 5-fold cross-validation as described above. External validation was performed using the independent Parkinson’s Progression Markers Initiative (PPMI) cohort to assess model generalizability.

Due to differences in data availability, certain biomarkers (triglycerides, TG) were not available in the external dataset. These features were retained and encoded as missing values to preserve the original data structure and avoid bias introduced by imputation. We additionally performed a sensitivity analysis in which TG was completely excluded from both the internal training context and the external validation dataset, while all other predictors and modeling procedures were kept unchanged.

Model performance was assessed using the area under the receiver operating characteristic curve (AUC) and classification accuracy. An optimal classification threshold was determined in the internal cohort using the Youden index (J = sensitivity + specificity − 1) and was then fixed and applied to the external cohort without further adjustment. Confusion matrices were used to summarize classification performance, and sensitivity and specificity were calculated accordingly.

To assess the contribution of baseline cognitive performance and determine whether the predictive performance of the model was driven predominantly by MoCA, two complementary ablation analyses were conducted. First, a MoCA-only TabPFN model was evaluated using MoCA as the sole predictor. Second, a MoCA-excluded model was evaluated using all predictors from the primary model except MoCA.

To quantify uncertainty, 95% confidence intervals (CIs) for AUC were estimated using bootstrap resampling with 1,000 iterations. All statistical analyses were performed using Python and R.

### 2.7 Interpretability: SHAP analysis

To interpret model predictions, SHapley Additive exPlanations (SHAP) were applied. SHAp values were calculated based on the internal validation cohort to assess feature importance. Global importance was quantified using mean absolute SHAP values, and results were visualized using SHAP summary plots to illustrate both the magnitude and direction of feature effects.

### 2.8 Model comparison

To assess the comparative performance of TabPFN, we additionally evaluated logistic regression and random forest classifiers using the same 10 predictor variables and identical five-fold stratified group cross-validation splits. Grouping was performed by patient identifier to prevent records from the same patient from appearing in both the training and validation sets. For logistic regression and random forest, missing values were imputed using the mean estimated exclusively from the corresponding training fold. Continuous variables were standardized for logistic regression. The logistic regression model used class-balanced weights, whereas the random forest comprised 100 trees with class-balanced weights. No explicit imputation was applied before TabPFN modeling. Model discrimination was assessed using the area under the receiver operating characteristic curve (AUC), and calibration was evaluated using calibration curves and the Brier score. AUC, Brier score, and accuracy were summarized across the five validation folds.

### 2.9 Longitudinal clinical analysis

To investigate temporal changes in clinical variables, a longitudinal paired analysis was conducted. For each patient, consecutive visits were paired (T and T1). Patients were categorized as converters (NCI → CI) or stable (NCI → NCI). Baseline differences between groups were assessed using analysis of covariance (ANCOVA) with adjustment for age. Longitudinal changes were quantified as annualized rates of change:


(ValueatT1−ValueatT)/time interval(years)

Differences between groups were compared using independent t-tests.

To further evaluate the longitudinal trajectories while making full use of all eligible repeated measurements, linear mixed-effects models were subsequently fitted. Time was calculated continuously in years from the first eligible cognitively normal visit. The models included time, cognitive-outcome group, and a time-by-group interaction as fixed effects, with a patient-specific random intercept to account for within-patient correlation. Baseline age, sex, diabetes, hypertension, and BMI were included as covariates. The time-by-group interaction was the primary parameter used to assess whether longitudinal rates of change differed between the Stable and Converter groups. Because TG and the residuals from the model using untransformed TG were markedly right-skewed, log-transformed TG was used in the primary mixed-effects analysis.

### 2.10 Lipidomics analysis

To explore the metabolic mechanisms underlying clinical findings, an independent cohort of patients with Parkinson’s disease (*n* = 30) was recruited for serum-based untargeted lipidomics analysis.

Lipidomic profiling was performed using an ultra-high performance liquid chromatography coupled with high-resolution mass spectrometry (UPLC-HRMS) platform. Quality control (QC) samples were used to monitor analytical stability, and features with high variability were excluded to ensure data reliability. Raw data were processed using standard workflows including peak detection, alignment, normalization, and annotation. Lipid species were categorized into functional classes based on established databases. Multivariate analyses (OPLS-DA) were conducted to assess global metabolic differences. Variable importance in projection (VIP) scores were used to identify influential lipid species. Univariate analysis was performed to evaluate differential expression, with fold change (FC), *p*-values, and false discovery rate (FDR) calculated. Lipids meeting predefined criteria (FC > 1.5 or FC < 0.67, VIP > 1 and FDR < 0.05) were considered significant. Further analyses were conducted at both lipid species and class levels, and correlation analyses were performed to link lipidomic features with clinical variables and model-derived risk probabilities.

This integrative analysis aimed to link clinical predictors identified by machine learning, particularly triglycerides (TG), with underlying lipidomic alterations at the molecular level.

This study was not registered as it is a retrospective observational study.

This study was conducted and reported in accordance with the TRIPOD+AI guidelines.

## 3 Results

### 3.1 Baseline characteristics

The baseline characteristics of the internal cohort are summarized in Table 1.

**Table 1:** Baseline characteristics of the internal cohort stratified by cognitive outcome.

Variable	Total	Stable (NCI→NCI)	Converter (NCI→CI)	*p* value	Age-adjusted *p* value
N	91	46	45		
Age	64.01 ± 8.25	61.59 ± 8.79	66.49 ± 6.92	0.004	
Male, *n* (%)	54 (59.3%)	28 (60.9%)	26 (57.8%)	0.833	
BMI (kg/m^2^)	24.49 ± 3.11	23.94 ± 3.06	25.03 ± 3.10	0.098	0.032
Education years	14.05 ± 3.18	14.54 ± 3.08	13.59 ± 3.26	0.263	
Disease duration	6.63 ± 4.37	6.54 ± 4.19	6.72 ± 4.60	0.845	
UPDRS-III (Off)	35.95 ± 15.55	32.74 ± 14.93	39.08 ± 15.69	0.070	0.547
MoCA	27.16 ± 1.50	27.81 ± 1.03	26.42 ± 1.60	<0.001	<0.001
Triglycerides (mmol/L)	1.09 ± 0.72	0.96 ± 0.40	1.23 ± 0.94	0.0902	0.032
Uric acid (μmol/L)	304.58 ± 70.95	298.90 ± 64.65	310.52 ± 77.29	0.442	0.388
Diabetes, *n* (%)	16 (17.6%)	8 (17.4%)	8 (17.8%)	1.000	
Hypertension, *n* (%)	29 (31.9%)	12 (26.1%)	17 (37.8%)	0.266	
T-to-T1 interval (months)	18.8 (10.8–28.8)	18.6 (11.3–29.7)	19.8 (10.4–28.2)	0.861	

Values are presented as mean ± SD or *n* (%), except for the T-to-T1 interval, which is presented as the median (interquartile range). *p* values were calculated using t-test or chi-square test. Age-adjusted *p* values were obtained using ANCOVA.

All patients were cognitively normal at baseline. During follow-up, 45 patients developed cognitive impairment (converters), while 46 remained cognitively stable. This design ensures that observed differences reflect predictors of future cognitive decline rather than consequences of existing impairment.

At baseline, converters were significantly older and had lower MoCA scores compared to stable individuals. In addition, triglyceride (TG) levels were higher in converters and remained significantly associated with cognitive decline after adjustment for age.

No significant differences were observed in sex, education level, disease duration, or other laboratory biomarkers between the two groups.

### 3.2 Model performance

The final model incorporated the following predictors: age at visit, years of education, disease duration, UPDRS-III score (off medication), MoCA score, body mass index (BMI), triglycerides (TG), uric acid (UA), levodopa responsiveness (MPI), and the predictive window (months).

The predictive performance of the model was evaluated using internal cross-validation and external validation.

In the internal cohort, the model achieved a mean area under the receiver operating characteristic curve (AUC) of 0.822 (95% CI: 0.709–0.889), indicating good discriminative ability (Figure 2A). Using the optimal threshold determined by the Youden index, the model demonstrated a sensitivity of 75.6% (95% CI 61.3–85.8%) and a specificity of 76.1% (95% CI 62.1–86.1%) (Figure 2B). Sensitivity analyses showed the model achieved moderate discrimination for first-recorded PD-MCI, whereas the PDD-specific estimate was less precise because of the limited number of PDD events (Supplementary Figure S1).

**Figure 2:**
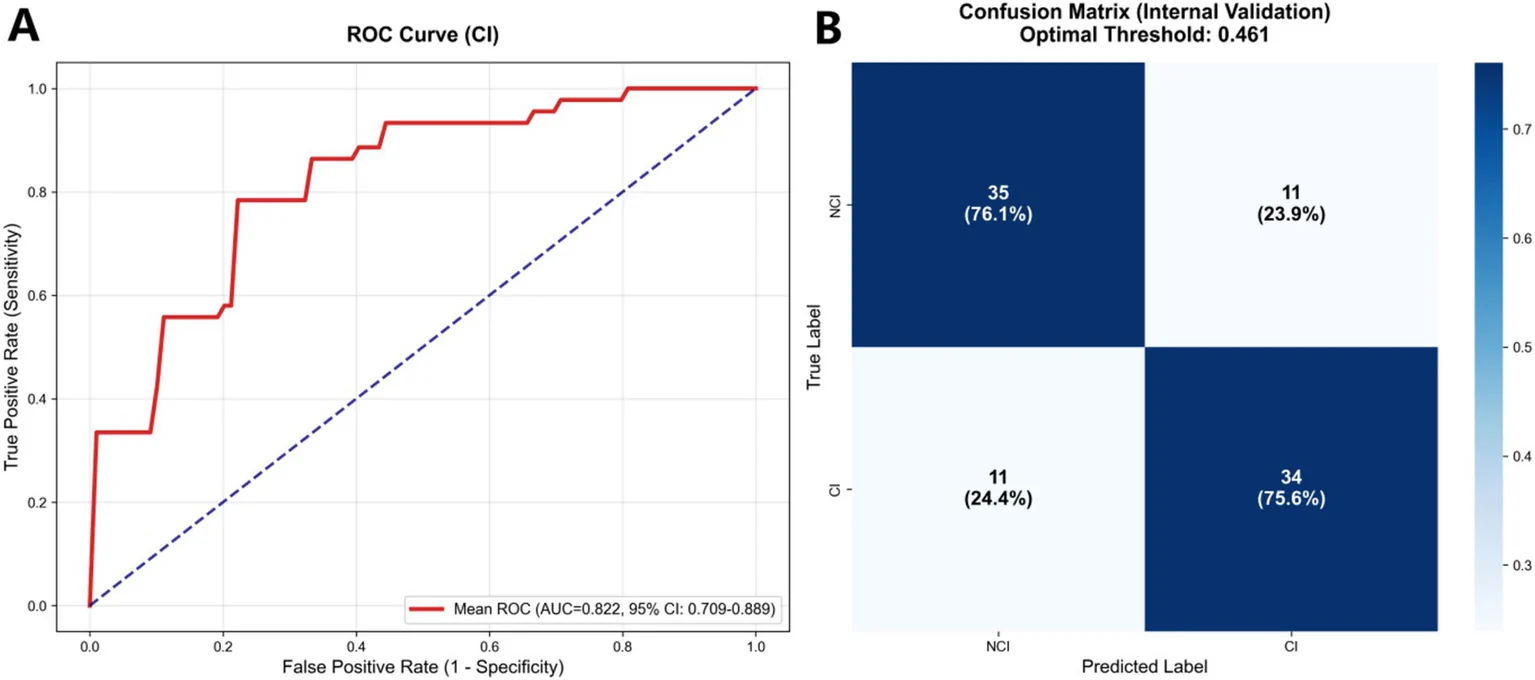
Model performance in internal validation cohort. **(A)** Receiver operating characteristic (ROC) curve of the model in the internal cohort, showing a mean AUC of 0.822 (95% CI: 0.709–0.889). **(B)** Confusion matrix for the internal validation using the optimal threshold determined by the Youden index (0.461).

In the MoCA-only ablation analysis, the model achieved an AUC of 0.742 (95% CI 0.599–0.826), which was numerically lower than that of the complete model (AUC = 0.822; Supplementary Figure S2). At the model-specific optimal threshold of 0.680, sensitivity was 44.4% and specificity was 93.5%. When MoCA was excluded and the remaining nine predictors were retained, the model achieved an AUC of 0.693 (95% CI 0.558–0.782; Supplementary Figure S3). At the model-specific optimal threshold of 0.443, sensitivity was 80.0% and specificity was 52.2%. Thus, removal of MoCA reduced discrimination, whereas MoCA alone did not reproduce the performance of the complete model. These findings indicate that MoCA was an important predictor, while the remaining clinical and biochemical variables provided complementary predictive information.

External validation was conducted using the independent PPMI cohort. The model achieved an AUC of 0.742 (95% CI: 0.695–0.783) (Figure 3A), providing evidence of external transportability. Applying the predefined threshold from the internal cohort, the model yielded a sensitivity of 75.0% (95% CI 57.9–86.7%) and a specificity of 68.9% (95% CI 65.9–71.8%) in the external dataset (Figure 3B). After TG was completely excluded in sensitivity analysis, the model was rerun using the remaining nine predictors, sensitivity remained unchanged at 75.0% (95% CI 57.9–86.7%), whereas specificity decreased to 64.4% (95% CI 61.3–67.4%). The external AUC also decreased slightly from 0.742 to 0.740 (Supplementary Figure S4). Thus, removing TG had little effect on the identification of patients who developed cognitive impairment but modestly reduced the model’s ability to correctly classify cognitively stable participants.

**Figure 3:**
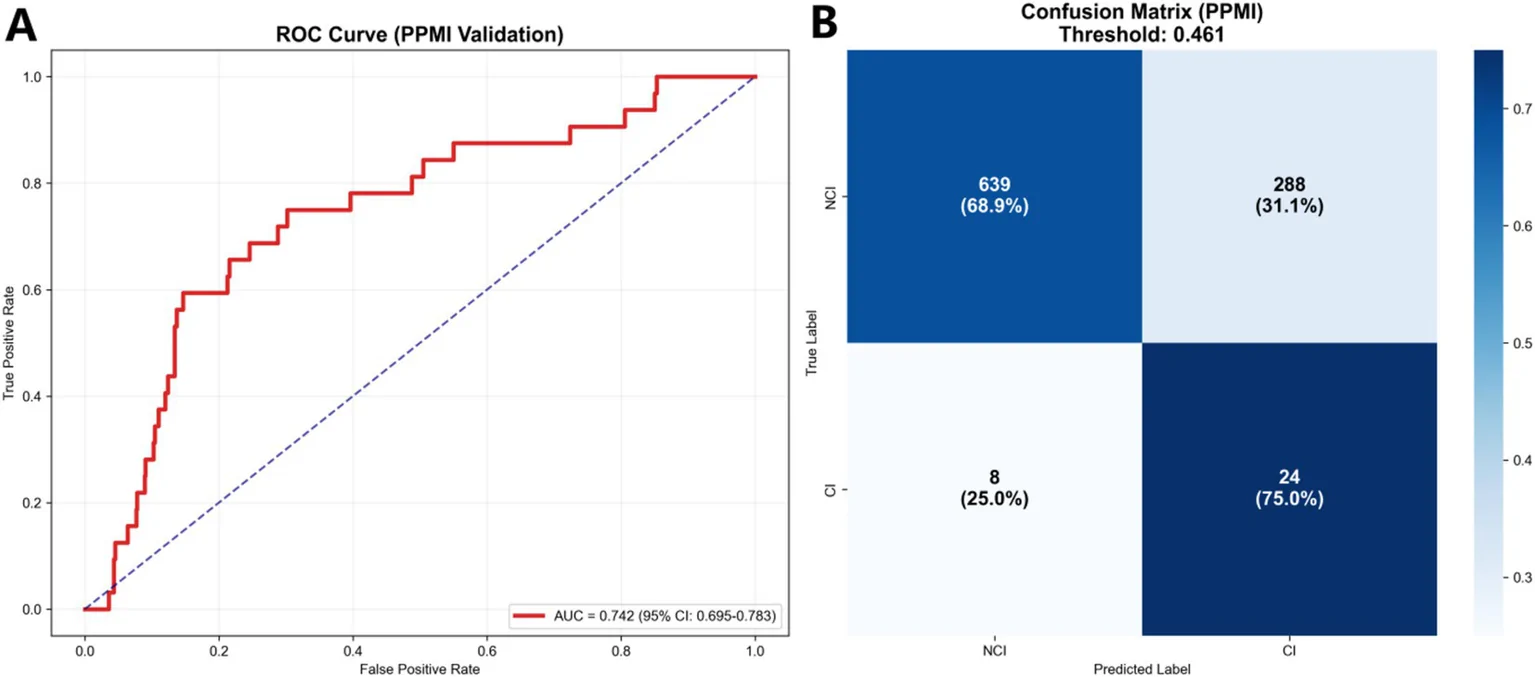
Model performance in external validation cohort. **(A)** ROC curve of the model in the external PPMI cohort, demonstrating an AUC of 0.742 (95% CI: 0.695–0.783). **(B)** Confusion matrix for the external validation using the same predefined threshold from the internal cohort.

The calibration curve demonstrated good agreement between predicted and observed probabilities, indicating satisfactory model calibration (Supplementary Figure S5).

Overall, the model showed stable performance across datasets, with consistently high sensitivity, suggesting its potential utility for early identification of patients at risk of cognitive impairment.

### 3.3 Model interpretability (SHAP analysis)

To interpret the model and identify key predictors, SHAP (SHapley Additive exPlanations) analysis was performed.

Global feature importance analysis showed that MoCA score and age were the most influential predictors, followed by triglyceride (TG) levels, education years, and BMI (Figure 4a). This indicates that, beyond established clinical factors, metabolic variables also contributed substantially to model predictions.

**Figure 4:**
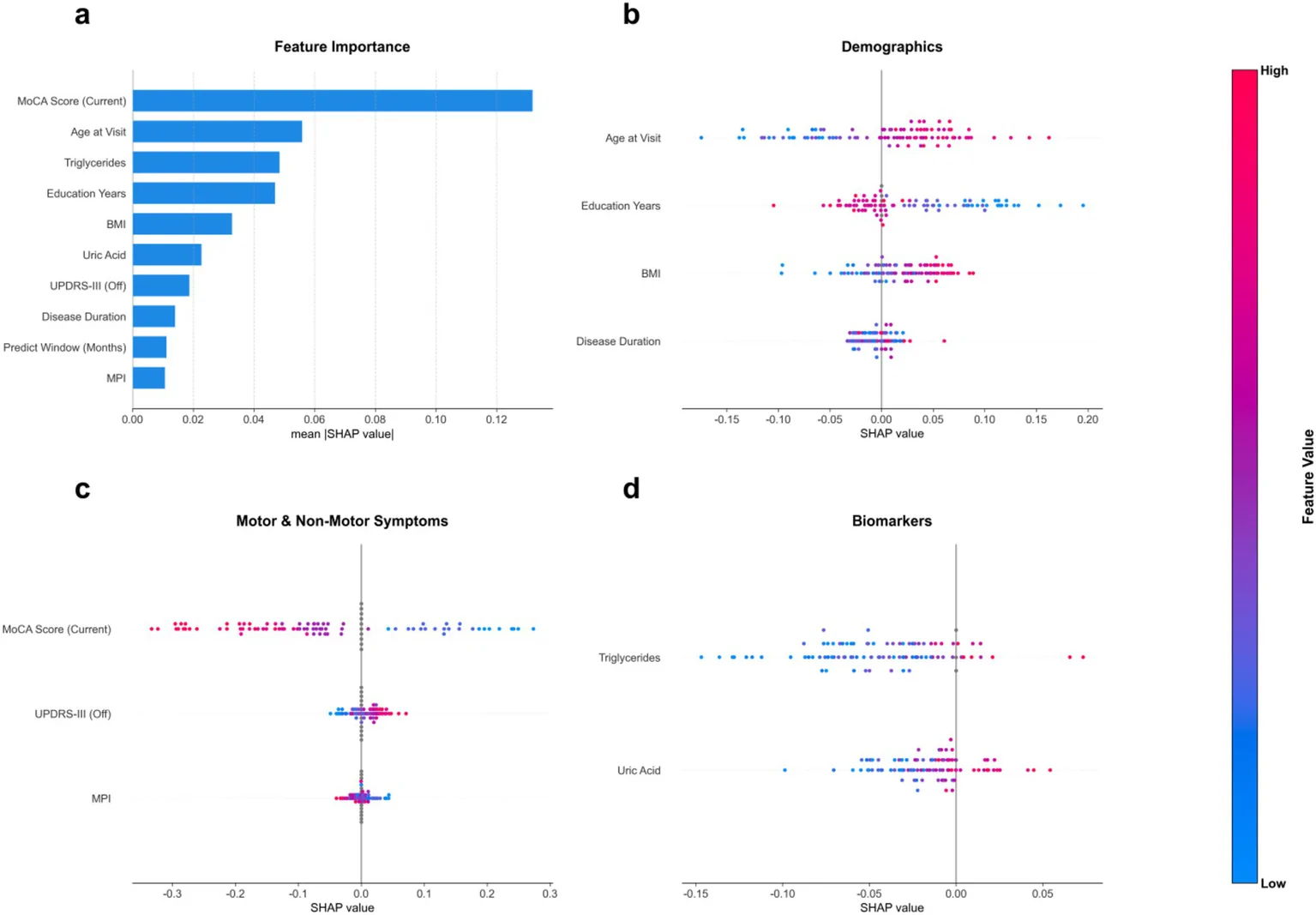
SHAP-based interpretation of the prediction model. **(a)** Global feature importance based on mean absolute SHA*p* values. **(b)** SHAP summary plot for demographic variables. **(c)** SHAP summary plot for motor and non-motor features. **(d)** SHAP summary plot for biochemical markers. Each point represents an individual sample. Red indicates higher feature values and blue indicates lower values. Positive SHAP values indicate increased predicted risk of cognitive impairment. Triglycerides (TG) consistently showed a positive contribution to risk, supporting its role as a key metabolic predictor. Levodopa responsiveness was quantified as the maximum percentage improvement (MPI) in UPDRS-III scores.

In the demographic domain, higher age was consistently associated with increased risk of cognitive impairment, while higher education levels were associated with a protective effect (Figure 4b).

Within motor and non-motor features, lower MoCA scores strongly contributed to higher predicted risk, whereas UPDRS-III and levodopa responsiveness (MPI) showed relatively modest effects (Figure 4c).

Notably, among biochemical markers, higher TG levels were associated with increased risk of cognitive impairment, demonstrating a consistent positive contribution across individuals (Figure 4d). In contrast, uric acid showed weaker effects.

These findings indicate that metabolic features, particularly TG, contribute to risk prediction beyond traditional clinical variables and play an important role in model-derived predictions, suggesting a potential link between metabolic alterations and cognitive impairment in Parkinson’s disease.

### 3.4 Model comparison

In the comparative analysis using the same 10 predictors and identical patient-grouped cross-validation splits, TabPFN achieved the highest mean AUC (0.822 ± 0.053), compared with random forest (0.787 ± 0.097) and logistic regression (0.728 ± 0.086). TabPFN also showed the lowest mean Brier score (0.182), compared with random forest (0.194) and logistic regression (0.221), with corresponding mean accuracies of 0.725, 0.701, and 0.660, respectively (Figure 5). These findings suggest that TabPFN provided numerically better discrimination and overall probabilistic accuracy in this dataset, although the relatively small sample size limits definitive conclusions regarding superiority.

**Figure 5:**
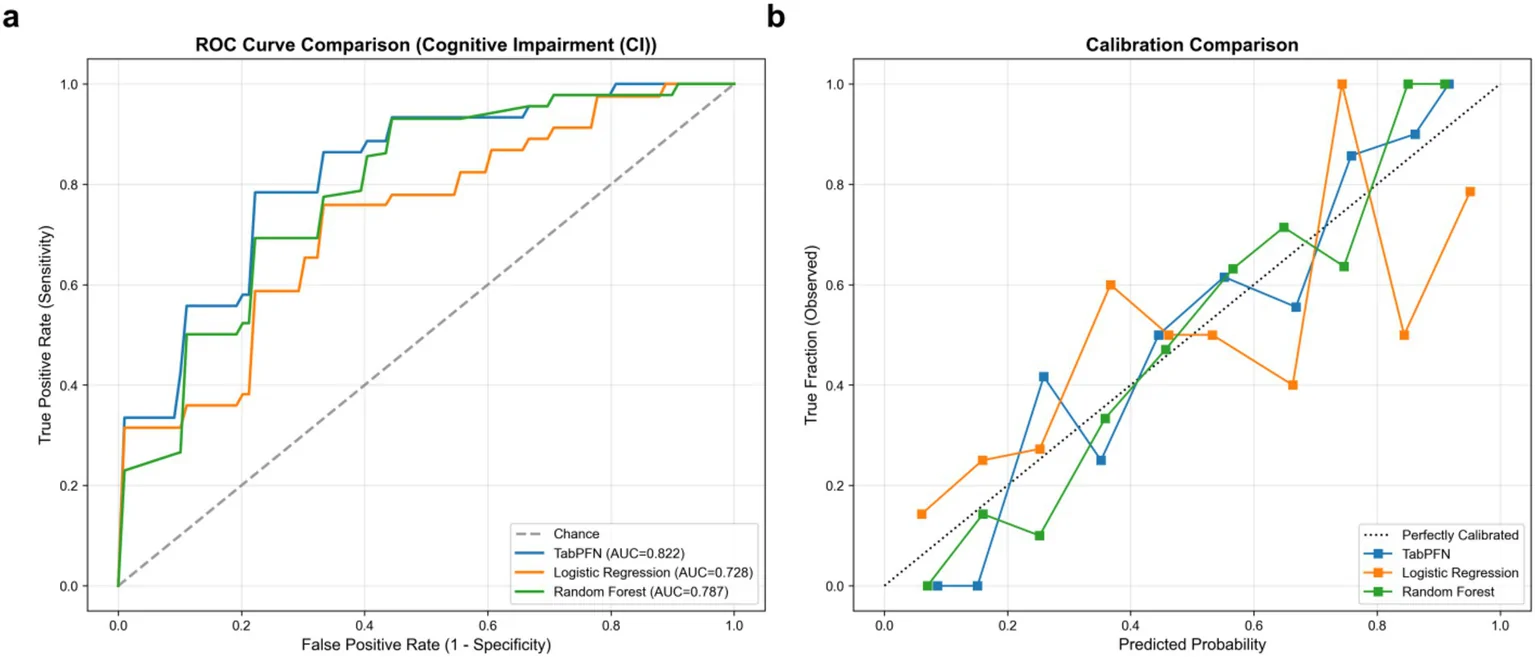
Comparison of TabPFN and conventional classification models in internal validation. All models used the same 10 predictors and identical five-fold stratified group cross-validation splits, with observations grouped by patient. Logistic regression and random forest used training-fold mean imputation, and predictors were standardized for logistic regression. **(a)** Mean receiver operating characteristic curves; reported AUC values represent the mean across validation folds. **(b)** Calibration curves based on pooled out-of-fold predictions. The diagonal line indicates perfect calibration.

### 3.5 Longitudinal changes between consecutive visits

To further investigate dynamic changes associated with cognitive decline, longitudinal analyses were performed comparing converters and cognitively stable individuals.

At baseline, converters exhibited higher triglyceride (TG) levels compared to stable individuals after adjustment for age. However, over time, TG levels showed a significant decline in converters, with a greater annualized decrease compared to the stable group (−0.149 vs. +0.09 per year, *p* = 0.009; Figure 6). Similarly, cognitive performance, as measured by MoCA, declined more rapidly in converters than in stable individuals (−3.616 vs. −0.357 per year, *p* < 0.001; Figure 6), reflecting clinical progression. In contrast, no significant differences were observed in the longitudinal changes of BMI, uric acid, or UPDRS-III scores between the two groups. These descriptive analyses suggested different TG trajectories between the two groups, with higher baseline TG levels and lower subsequent values among converters.

**Figure 6:**
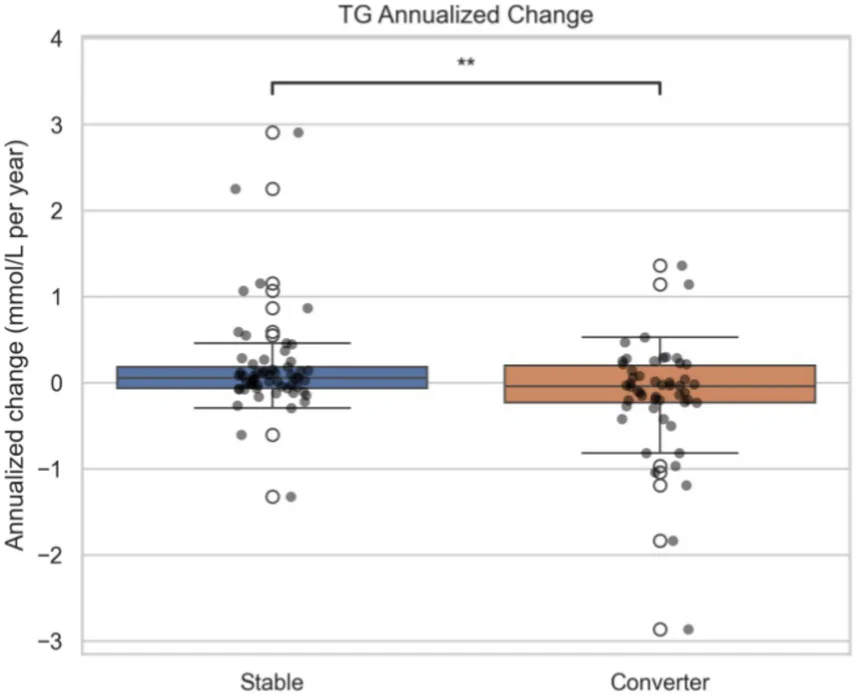
Longitudinal changes in triglycerides (TG). Annualized rate of change in TG levels between converters and stable individuals. TG levels decreased significantly in converters, while remaining stable or slightly increased in the stable group (*p* = 0.009). Each point represents an individual patient. Boxes indicate median and interquartile range.

The initial comparison of annualized change rates provided a descriptive assessment of differences between the Stable and Converter groups. To account for unequal follow-up intervals, repeated within-patient measurements, and potential confounders, we subsequently performed a linear mixed-effects analysis (Table 2).

**Table 2:** Linear mixed-effects model of longitudinal TG.

Variable	Beta	95% CI lower	95% CI upper	*p* value
Intercept	−0.024	−0.276	0.228	0.851
Time (years)	0.063	−0.018	0.144	0.128
Converter group	0.149	−0.136	0.434	0.305
Time × Converter interaction	−0.146	−0.248	−0.044	0.005
Baseline age	−0.005	−0.021	0.01	0.5
Sex	0.002	−0.256	0.26	0.987
Diabetes	−0.024	−0.368	0.32	0.891
Hypertension	−0.078	−0.345	0.189	0.566
Baseline BMI	0.049	0.008	0.091	0.019

The model was adjusted for baseline age, sex, diabetes, hypertension, and BMI. The Stable group was the reference: the time coefficient represents its slope, the Converter coefficient represents the baseline group difference, and the time-by-Converter interaction represents the difference in slopes. The Converter-group slope equals the sum of the time and interaction coefficients.

In the fully adjusted model of log-transformed TG, the estimated annual change was 6.5% in the Stable group (95% CI, −1.8 to 15.5%; *p* = 0.128) and −8.0% in the Converter group (95% CI, −13.6 to −2.1%; *p* = 0.009). The time-by-group interaction was significant (β = −0.146, 95% CI, −0.248 to −0.044; *p* = 0.005), indicating that the longitudinal TG trajectory differed between groups after adjustment for baseline age, sex, diabetes, hypertension, and BMI. We also conducted a sensitivity analysis restricted to cognitively unimpaired visits before conversion. The group-by-time interaction remained significant (β = −0.154, 95% CI − 0.278 to −0.030; *p* = 0.015), suggesting that the differential TG trajectory was already detectable before the first visit documenting cognitive impairment. However, only 10 converters had at least two eligible pre-conversion TG measurements; therefore, this finding should be interpreted cautiously (Supplementary Table S4).

### 3.6 Lipidomics analysis

To explore the molecular lipid profiles associated with cognitive status in PD, we performed an untargeted lipidomics analysis in an independently recruited, cross-sectional cohort. This analysis was exploratory and was not designed to validate the longitudinal TG trajectory observed in the retrospective clinical cohort.

The independent lipidomics cohort included 20 participants with CI and 10 participants with NCI. Participants with CI tended to be older than those with NCI (64.5 ± 11.4 vs. 57.1 ± 9.0 years; *p* = 0.068), whereas sex distribution (male: 50.0% vs. 60.0%; *p* = 0.709) and disease duration (8.7 ± 4.3 vs. 7.0 ± 4.1 years; *p* = 0.339) did not differ significantly. All participants with available medication records received levodopa-containing therapy, and no clear differences in the recorded antiparkinsonian medication classes were observed between groups. None of the participants used lipid-lowering therapy. However, given the small sample size, the absence of statistically significant differences should not be interpreted as evidence of complete covariate balance. Given the age imbalance, lipid-level comparisons were adjusted for age using overlap propensity-score weighting. We subsequently examined the association between routine biochemical TG measurements and total TG abundance derived from lipidomic profiling. A strong correlation was observed between the two measurements (R = 0.94; Figure 7), indicating concordance between routine biochemical TG measurements and lipidomics-derived total TG abundance.

**Figure 7:**
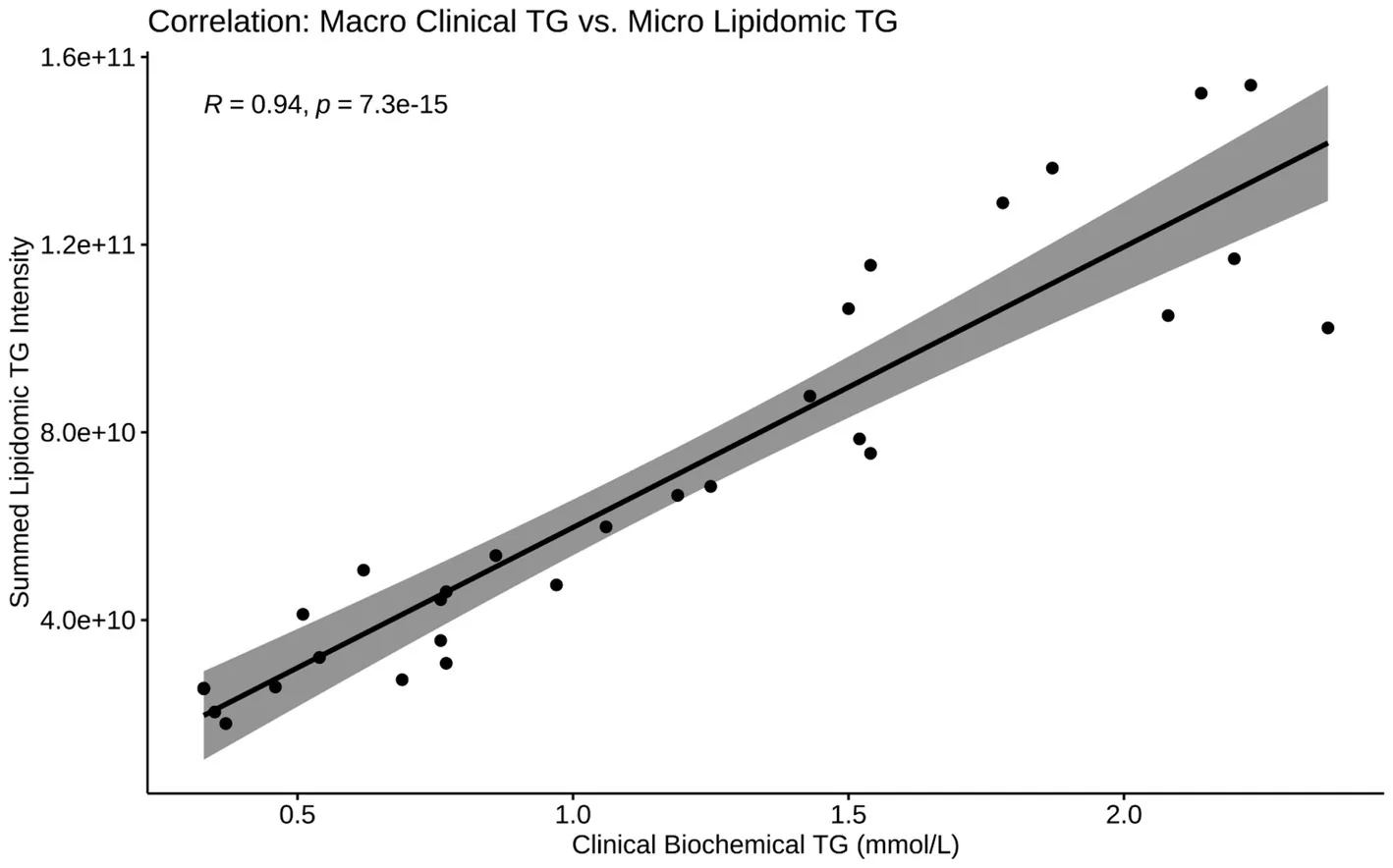
Correlation between clinical triglyceride levels and lipidomic triglyceride abundance in patients with Parkinson’s disease. Correlation between clinical triglyceride (TG) levels and total lipidomic TG abundance, demonstrating strong consistency between routine biomarkers and molecular lipid profiles.

At the lipid-class level, total TG abundance did not differ significantly between the CI and NCI groups (*p* = 0.65; Figure 8). Exploratory species-level analysis nevertheless identified several lipid species that differed between groups according to the prespecified criteria of VIP > 1, FDR < 0.05, and fold change > 1.5 or < 0.67 (Figure 9). Most showed lower abundance in the CI group, including several ceramide (Cer), sphingomyelin (SM), and phosphatidylcholine (PC) species.

**Figure 8:**
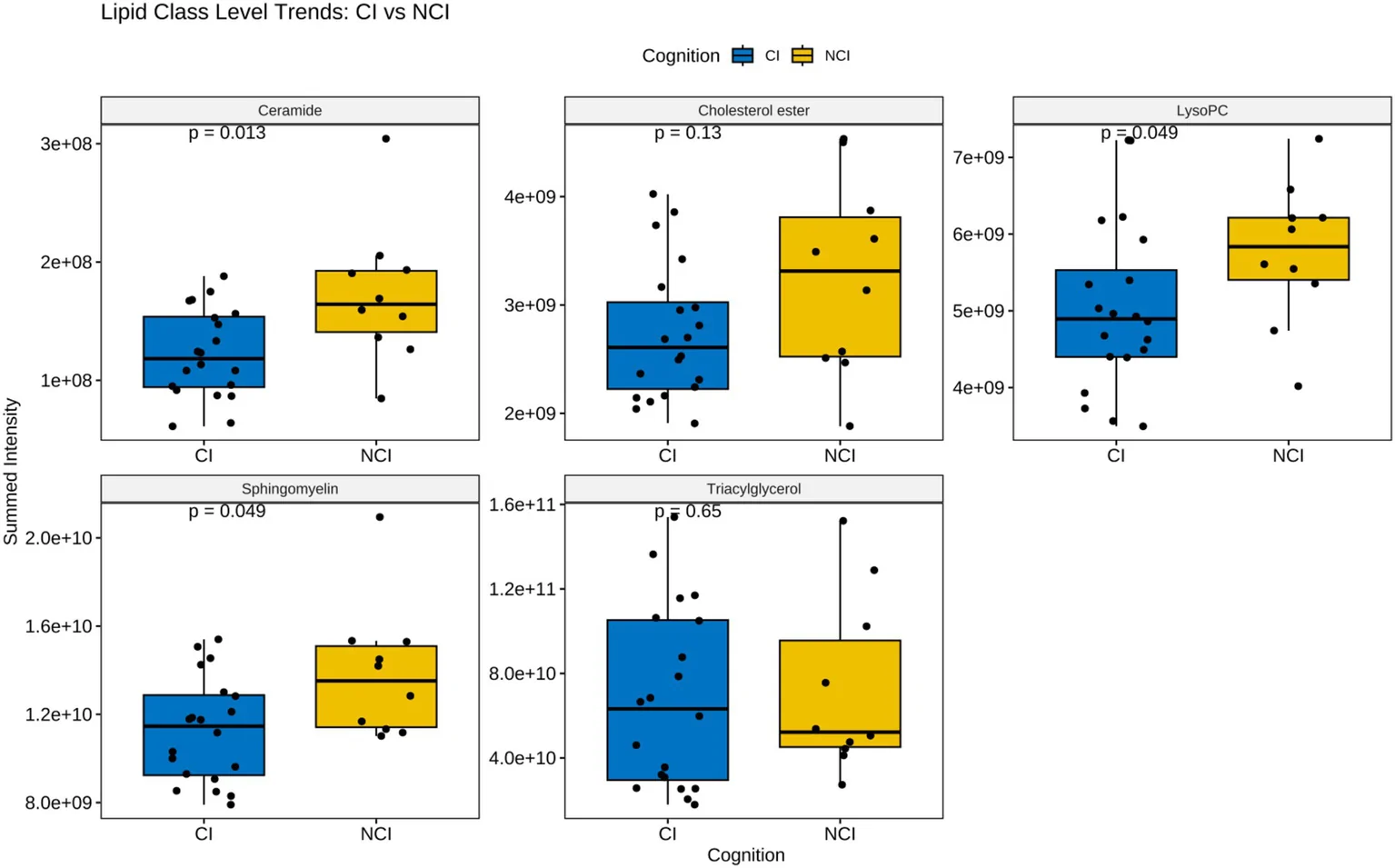
Comparison of lipid class–level abundances between PD patients with cognitive impairment (CI) and normal cognition (NCI). Class-level comparison of major lipid categories, including ceramides, cholesterol ester, triacylglycerol, sphingomyelins, and lysophosphatidylcholines (LysoPC), between groups.

**Figure 9:**
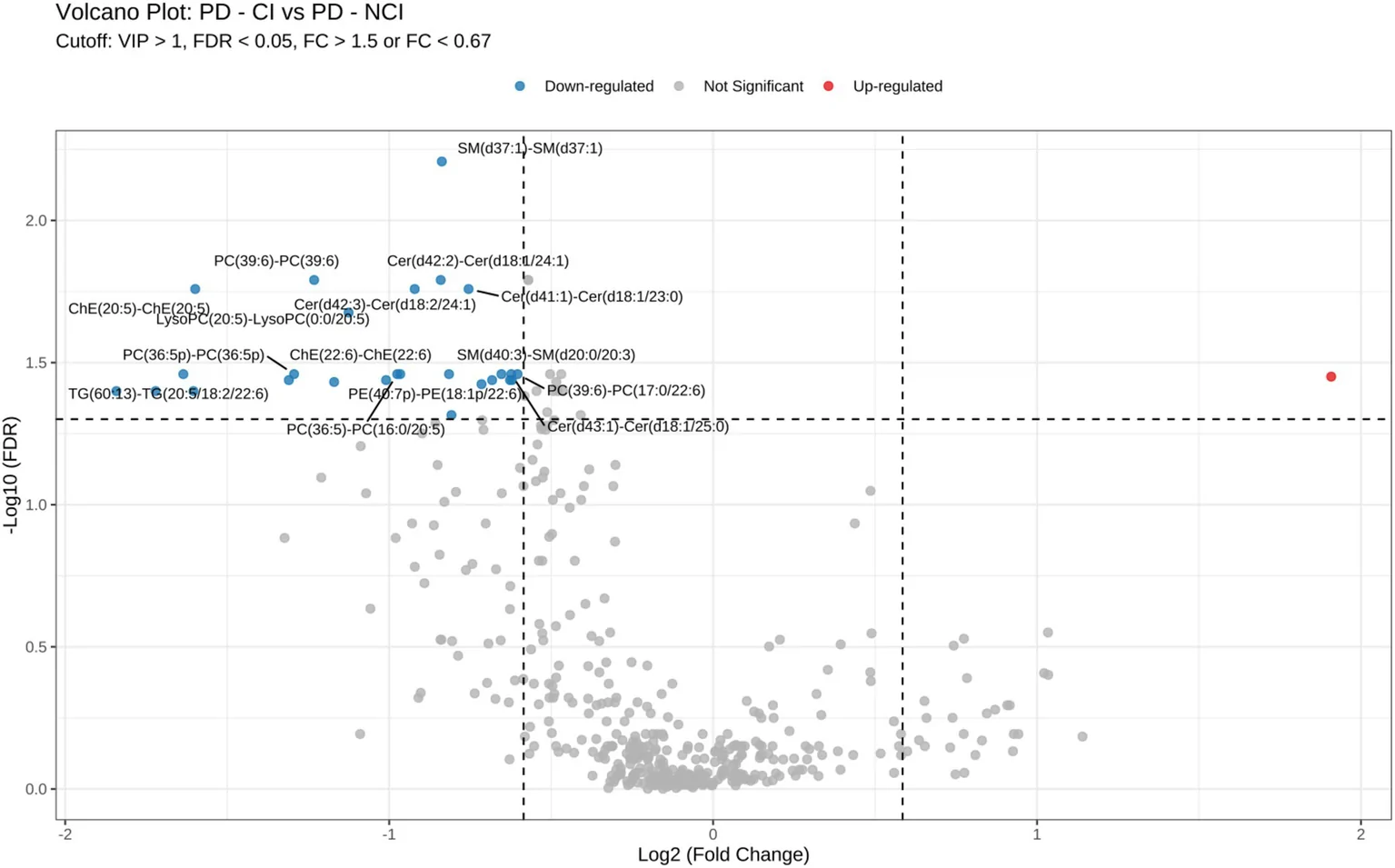
Volcano plot of differential lipid species between PD patients with cognitive impairment (CI) and those with normal cognition (NCI). Volcano plot showing differentially expressed lipid species between cognitively impaired (CI) and cognitively normal (NCI) individuals. Significant lipids were defined as VIP > 1, FDR < 0.05, and fold change > 1.5 or < 0.67.

Among the TG species meeting these criteria, several species with lower abundance in the CI group contained polyunsaturated fatty-acid chains. These findings suggest that cognitive-status-associated lipid differences may occur at the molecular-species level even when aggregate class-level abundance is similar. Because this independent cohort was small and cross-sectional, these findings should be considered exploratory and hypothesis-generating; confirmation in larger prospective cohorts with repeated lipidomic measurements is required.

## 4 Discussion

In this study, we developed and externally validated a machine learning–based predictive model for future cognitive impairment in patients with Parkinson’s disease using routinely available clinical data. The model demonstrated good discriminative performance, with an AUC of 0.822 in internal validation and 0.742 in the independent PPMI cohort, supporting its robustness and generalizability across populations. In exploratory benchmark comparisons using the same predictors and cross-validation framework, TabPFN showed higher discrimination than logistic regression and random forest, although the difference relative to random forest was modest. These findings suggest that a pretrained tabular model may be useful for risk prediction in small clinical datasets, but they should not be interpreted as establishing the superiority of TabPFN without evaluation in larger cohorts.

The prediction window represented the interval between the assessment at which predictor variables were obtained and the future visit at which cognitive status was evaluated. Including this variable allowed the estimated risk to be interpreted in relation to a specified future timeframe, because the probability of cognitive impairment is expected to increase with time. Importantly, the T-to-T1 interval did not differ significantly between converters and stable participants, and the prediction window alone showed essentially no discriminatory ability (AUC = 0.510). Therefore, the discrimination of the complete model was unlikely to be explained solely by systematic differences in follow-up duration between outcome groups. Nevertheless, the present analysis predicts cognitive status at the evaluated future observation window rather than explicitly modeling event times or censoring.

External validation provided evidence that the model retained moderate discrimination in an independently collected cohort, despite differences in clinical setting and data availability. However, this should be interpreted as preliminary evidence of transportability rather than proof of broad generalizability. TG measurements were largely unavailable in PPMI and were therefore encoded as missing in the primary external analysis. Sensitivity analysis shows that the model is largely independent of the availability of TG in the PPMI dataset. However, because TG was largely unavailable in the external cohort, the external analysis cannot independently validate the specific predictive contribution of TG.

Age and MoCA were the most influential predictors, consistent with their established associations with cognitive impairment in PD. TG also contributed to model predictions, with higher baseline values generally associated with higher predicted risk. This finding is consistent with previous reports associating elevated serum TG or related metabolic indices with cognitive impairment in PD (Cao et al., 2025; Huang et al., 2018). Nevertheless, SHA*p* values describe how a feature contributes to predictions made by the fitted model and do not establish an independent causal effect. The TG finding should therefore be interpreted as a predictive association that warrants further investigation rather than evidence that TG directly contributes to cognitive decline.

The longitudinal clinical analysis provided additional information about changes in routine TG measurements. The annualized two-visit comparisons showed a greater reduction in TG among converters than among stable participants. Because annualized changes based on two observations can be affected by unequal intervals and measurement variability, we further applied a linear mixed-effects model incorporating all available TG measurements and accounting for within-patient correlation. After log transformation of TG and adjustment for baseline age, sex, BMI, diabetes, and hypertension, the group-by-time interaction remained significant (β = −0.146, 95% CI − 0.248 to −0.044; *p* = 0.005), indicating that the temporal slope differed between converters and stable participants. Another sensitivity analysis restricted to visits at which participants remained cognitively normal before conversion produced a similar group-by-time interaction (β = −0.154, 95% CI − 0.278 to −0.030; *p* = 0.015). This reduces the possibility that the observed difference was driven entirely by measurements obtained at the visit when cognitive impairment was first documented. Nevertheless, converters had higher baseline TG levels, and regression to the mean cannot be completely excluded. Moreover, the converter-specific slope was not independently significant in the preconversion analysis; therefore, the most defensible interpretation is that TG trajectories differed between groups, rather than that a definitive decline was demonstrated within converters alone. Prospective studies with standardized sampling intervals and repeated preconversion measurements are needed to characterize this temporal pattern more precisely.

The independent lipidomics analysis provided exploratory information about lipid profiles associated with cognitive status. Routine biochemical TG concentrations were strongly correlated with lipidomics-derived total TG abundance (R = 0.94), demonstrating concordance between the two measurement approaches. At the lipid-class level, however, total TG abundance did not differ significantly between CI and NCI participants. Species-level analyses identified differences in several individual lipid species, including TG species containing polyunsaturated fatty-acid chains and species belonging to ceramide, sphingomyelin, and phosphatidylcholine classes. These findings suggest that cognitive-status-associated differences may occur at the molecular-species level even when aggregate class-level abundance is similar. The lipidomics findings should nevertheless be interpreted cautiously. The cohort was independently recruited, small, and cross-sectional. Although the groups showed no statistically significant differences in sex, disease duration, or recorded antiparkinsonian medication classes, it does not demonstrate complete balance in a sample of this size. Furthermore, the correlation between routine and lipidomics-derived TG measurements establishes measurement concordance but does not validate the elevated-then-declining TG trajectory observed in the clinical cohort. Similarly, species-level differences cannot demonstrate temporal lipid remodeling without repeated lipidomic measurements.

Previous studies have reported inconsistent associations between circulating lipids and PD outcomes, with both higher and lower lipid levels being associated with disease severity or cognitive impairment (Fu et al., 2020; Huang et al., 2018; Zhang et al., 2022). Variation in disease stage, nutritional status, metabolic comorbidities, medication exposure, sampling conditions, and study design may contribute to these discrepancies. Our findings raise the hypothesis that the association between TG and cognitive status may vary over the disease course, but the current data do not establish a stage-dependent biological mechanism. Explanations involving insulin resistance, altered lipid transport, mitochondrial dysfunction, inflammation, or impaired lipid synthesis remain speculative (Cao et al., 2025; Fernandes et al., 2023; Fu et al., 2020; Qin et al., 2025; Truong et al., 2025; Zhang et al., 2022). Confirmation would require prospective studies combining repeated routine TG measurements with repeated lipidomic profiling and relevant metabolic markers.

From a clinical perspective, the use of routinely available variables may facilitate accessible risk stratification without requiring cerebrospinal fluid biomarkers, advanced imaging, genetic testing, or other specialized measurements. The model may therefore be useful for identifying patients who warrant closer cognitive monitoring. However, prospective multicenter validation, assessment of calibration across clinical settings, and evaluation of clinical utility are required before implementation.

The relatively high conversion proportion in the internal cohort should also be considered when interpreting clinical applicability. The cohort consisted of repeatedly admitted patients at a single tertiary neurological center and may consequently have included individuals with greater disease complexity, more advanced disease, referral enrichment, or more intensive clinical surveillance than typical outpatient PD cohorts. These factors may have increased the probability that cognitive impairment was documented during follow-up. Accordingly, the observed conversion proportion should not be interpreted as an estimate of the incidence of cognitive impairment in the broader PD population. Although external validation supports some transportability of the prediction framework, differences in disease spectrum and outcome prevalence may affect calibration and predictive values when the model is applied in other settings.

PD-MCI and PDD were pooled as a composite cognitive-impairment outcome to provide a clinically relevant screening endpoint and to maintain adequate statistical stability in the small development cohort. This approach is appropriate for identifying patients at risk of any clinically important cognitive deterioration but does not imply that PD-MCI and PDD are clinically or biologically equivalent. Sensitivity analyses separating the two outcomes produced less precise estimates, particularly for PDD, because of the reduced number of events. Therefore, the current model should be interpreted as predicting a composite cognitive-impairment outcome rather than distinguishing the stage or severity of cognitive decline.

## 5 Limitations

Several limitations should be acknowledged. First, the development cohort was relatively small, retrospective, and restricted to repeatedly admitted patients at a single tertiary center. Referral and selection patterns may therefore limit model calibration and generalizability to outpatient or community PD populations. The recorded outcome date represented the first clinical documentation of cognitive impairment rather than its biological onset. In addition, the primary binary outcome pooled PD-MCI and PDD, and the prediction framework did not explicitly model event times or censoring. Because TG was largely unavailable in PPMI, external validation assessed the overall prediction framework but could not independently confirm the predictive contribution of TG. Second, annualized changes derived from selected visit pairs are sensitive to irregular follow-up and measurement variability. Linear mixed-effects modeling using all available TG measurements and the preconversion sensitivity analysis reduced, but did not eliminate, concerns regarding regression to the mean and residual confounding. Finally, the independent lipidomics cohort was small and cross-sectional; these results should therefore be considered exploratory and cannot establish temporal lipid remodeling or a mechanistic relationship between lipid alterations and cognitive decline.

## 6 Conclusion

In conclusion, a TabPFN-based model using routinely collected clinical and biochemical variables showed promising discrimination for future cognitive impairment in PD in internal and external evaluations. TG contributed to prediction in the internal cohort, and longitudinal analyses identified different TG trajectories between converters and stable participants. The independent cross-sectional lipidomics analysis identified cognitive-status-associated differences in individual lipid species but did not validate the longitudinal TG pattern or establish an underlying mechanism. These findings support further prospective, multicenter investigation of routinely available biomarkers for cognitive risk stratification and of the temporal relationship between lipid metabolism and cognitive decline in PD.

## Data Availability

The raw data supporting the conclusions of this article will be made available by the authors, without undue reservation.
