# Supplementary material for: Prediction of cognitive impairment in Parkinson’s disease using TabPFN: an integrative analysis of clinical data, longitudinal trajectories, and lipidomics
Source: Front Aging Neurosci. 2026 Sep 30;18:1905374. doi: 10.3389/fnagi.2026.1905374 (PMC13662187; doi:10.3389/fnagi.2026.1905374)
Supplement: Supplementary file 1 [file Supplementary_file_1.DOCX]

| Variable | Category | Missing (%) | Description |
| --- | --- | --- | --- |
| Age | Demographic | 0 | Age at clinical visit (years) |
| Sex | Demographic | 0 | Sex (male/female) |
| Education years | Demographic | 5.49 | Years of education |
| Duration years | Clinical | 0 | Disease duration (years) |
| Updrs iii off | Clinical | 13.19 | UPDRS-III (off medication) |
| MPI | Clinical | 15.38 | Levodopa responsiveness (MPI) |
| Moca | Cognitive | 2.2 | Montreal Cognitive Assessment score |
| Predict Window Months | Time | 0 | Prediction interval (months) |
| BMI | Clinical | 2.2 | Body mass index |
| TG | Laboratory | 3.3 | Triglycerides |
| TC | Laboratory | 16.48 | Total cholesterol |
| HDL-C | Laboratory | 16.48 | HDL cholesterol |
| LDL-C | Laboratory | 16.48 | LDL cholesterol |
| ApoA1 | Laboratory | 2.2 | Apolipoprotein A1 |
| ApoB | Laboratory | 2.2 | Apolipoprotein B |
| UA | Laboratory | 1.1 | Uric acid |
| Hcy | Laboratory | 2.2 | Homocysteine |
| NLR | Laboratory | 26.37 | Neutrophil-to-lymphocyte ratio |
| WBC | Laboratory | 0 | White blood cell count |
| ALT | Laboratory | 0 | Alanine aminotransferase |
| AST | Laboratory | 0 | Aspartate aminotransferase |
| ALB | Laboratory | 0 | Albumin |
| Urea | Laboratory | 16.48 | Blood urea nitrogen |
| Cr | Laboratory | 25.27 | Creatinine |
| eGFR | Laboratory | 25.27 | Estimated glomerular filtration rate |
| Glu | Laboratory | 2.2 | Glucose |
| FT3 | Laboratory | 14.29 | Free triiodothyronine |
| FT4 | Laboratory | 14.29 | Free thyroxine |
| TSH | Laboratory | 14.29 | Thyroid-stimulating hormone |
| Anxiety | Non-motor | 0 | Anxiety symptoms |
| Depression | Non-motor | 0 | Depression symptoms |
| Hallucination | Non-motor | 0 | Hallucinations |
| Constipation | Non-motor | 0 | Constipation |
| Hyposmia | Non-motor | 0 | Hyposmia |
| Orthostatic hypotension | Non-motor | 0 | Orthostatic hypotension |
| Daytime sleepiness | Non-motor | 0 | Daytime sleepiness |
| RBD | Non-motor | 0 | REM sleep behavior disorder |
| Hypertension | Comorbidity | 0 | Hypertension |
| Diabetes | Comorbidity | 0 | Diabetes |
| Smoking history | Lifestyle | 0 | Smoking history |
| Family history | Lifestyle | 0 | Family history |

**Table S1.** Full list of candidate predictors used for model development, including data availability, missingness, and variable descriptions.

Missing (%) was calculated as the proportion of missing values relative to the total number of observations. Variables were categorized based on clinical relevance.

| Variable | Total | Stable (NCI→NCI) | Converter (NCI→CI) | P value | Age-adjusted P value |
| --- | --- | --- | --- | --- | --- |
| N | 91 | 46 | 45 |  |  |
| Age (years) | 64.01 ± 8.25 | 61.59 ± 8.79 | 66.49 ± 6.92 | 0.004 | — |
| Sex (Male, %) | 54 (59.3%) | 28 (60.9%) | 26 (57.8%) | 0.8325 | — |
| Education (years) | 14.05 ± 3.18 | 14.54 ± 3.08 | 13.59 ± 3.26 | 0.2633 | — |
| Disease duration (years) | 6.63 ± 4.37 | 6.54 ± 4.19 | 6.72 ± 4.60 | 0.8454 | — |
| UPDRS Part III (off) | 35.95 ± 15.55 | 32.74 ± 14.93 | 39.08 ± 15.69 | 0.07 | 0.5467 |
| MoCA score | 27.16 ± 1.50 | 27.81 ± 1.03 | 26.42 ± 1.60 | <0.001 | 0.0001 |
| Body mass index (BMI) | 24.49 ± 3.11 | 23.94 ± 3.06 | 25.03 ± 3.10 | 0.0979 | 0.0321 |
| Triglycerides (TG) | 1.09 ± 0.72 | 0.96 ± 0.40 | 1.23 ± 0.94 | 0.0902 | 0.0322 |
| Homocysteine (Hcy) | 18.04 ± 15.21 | 16.68 ± 9.04 | 19.49 ± 19.83 | 0.3981 | 0.4468 |
| Uric acid (UA) | 304.58 ± 70.95 | 298.90 ± 64.65 | 310.52 ± 77.29 | 0.4421 | 0.3883 |
| Levodopa responsiveness (MPI) | 0.43 ± 0.17 | 0.45 ± 0.18 | 0.41 ± 0.15 | 0.2228 | 0.396 |
| NLR | 2.01 ± 0.73 | 2.02 ± 0.58 | 2.00 ± 0.87 | 0.894 | 0.4028 |
| WBC | 5.62 ± 1.60 | 5.79 ± 1.48 | 5.44 ± 1.73 | 0.3811 | 0.5948 |
| TC | 4.21 ± 0.91 | 4.17 ± 0.79 | 4.25 ± 1.04 | 0.7405 | 0.6459 |
| HDL-C | 1.42 ± 0.45 | 1.41 ± 0.29 | 1.44 ± 0.57 | 0.8304 | 0.8102 |
| LDL-C | 2.41 ± 0.77 | 2.42 ± 0.73 | 2.39 ± 0.83 | 0.9034 | 0.7663 |
| ApoA1 | 1.36 ± 0.25 | 1.37 ± 0.24 | 1.34 ± 0.26 | 0.5752 | 0.5794 |
| ApoB | 0.77 ± 0.21 | 0.75 ± 0.19 | 0.79 ± 0.24 | 0.4279 | 0.2997 |
| ALT | 15.12 ± 8.43 | 16.42 ± 7.74 | 13.70 ± 9.04 | 0.1923 | 0.2022 |
| AST | 18.68 ± 6.40 | 20.42 ± 7.23 | 16.83 ± 4.84 | 0.0187 | 0.0143 |
| ALB | 40.26 ± 3.82 | 40.36 ± 2.54 | 40.16 ± 4.87 | 0.8323 | 0.2217 |
| Urea | 8.38 ± 21.65 | 5.60 ± 1.59 | 10.87 ± 29.78 | 0.3583 | 0.1128 |
| Creatinine | 63.46 ± 13.88 | 64.88 ± 15.26 | 62.14 ± 12.62 | 0.4865 | 0.2801 |
| eGFR | 102.67 ± 16.49 | 106.97 ± 11.75 | 98.10 ± 19.52 | 0.0286 | 0.3768 |
| Glucose | 5.25 ± 1.38 | 4.87 ± 0.54 | 5.72 ± 1.89 | 0.1089 | 0.1371 |
| FT3 | 4.48 ± 0.52 | 4.57 ± 0.58 | 4.39 ± 0.45 | 0.1646 | 0.5159 |
| FT4 | 11.19 ± 1.84 | 11.12 ± 2.07 | 11.27 ± 1.61 | 0.7486 | 0.901 |
| TSH | 2.21 ± 2.29 | 1.86 ± 1.53 | 2.58 ± 2.85 | 0.2085 | 0.3484 |
| Hallucinations (Yes, %) | 12 (13.2%) | 7 (15.2%) | 5 (11.1%) | 0.7582 | — |
| Depression (Yes, %) | 34 (37.4%) | 20 (43.5%) | 14 (31.1%) | 0.2801 | — |
| Anxiety (Yes, %) | 41 (45.1%) | 22 (47.8%) | 19 (42.2%) | 0.6751 | — |
| Constipation (Yes, %) | 65 (71.4%) | 31 (67.4%) | 34 (75.6%) | 0.4877 | — |
| Hyposmia (Yes, %) | 58 (63.7%) | 31 (67.4%) | 27 (60.0%) | 0.5173 | — |
| Orthostatic hypotension (Yes, %) | 41 (45.1%) | 25 (54.3%) | 16 (35.6%) | 0.0928 | — |
| Daytime sleepiness (Yes, %) | 7 (8.1%) | 4 (9.1%) | 3 (7.1%) | 1 | — |
| RBD (Yes,%) | 56 (61.5%) | 29 (63.0%) | 27 (60.0%) | 0.831 | — |
| Hypertension (Yes, %) | 29 (32.2%) | 12 (26.1%) | 17 (38.6%) | 0.2606 | — |
| Diabetes (Yes, %) | 16 (17.8%) | 8 (17.4%) | 8 (18.2%) | 1 | — |
| Smoking history (Yes, %) | 15 (18.1%) | 7 (17.1%) | 8 (19.0%) | 1 | — |
| Family history (Yes, %) | 11 (12.9%) | 7 (15.9%) | 4 (9.8%) | 0.5232 | — |

**Table S2.** Extended baseline characteristics comparing converters and stable individuals.

Continuous variables are presented as mean ± standard deviation (SD), and categorical variables are presented as counts (percentages). P values were calculated using appropriate statistical tests. Age-adjusted P values were obtained using regression models with age as a covariate. CI, cognitive impairment; NCI, no cognitive impairment.

| Variable | Total | Stable (NCI→NCI) | Converter (NCI→CI) | P value | Age-adjusted P value |
| --- | --- | --- | --- | --- | --- |
| N | 91 | 46 | 45 |  |  |
| Age | 65.80 ± 8.10 | 63.41 ± 8.85 | 68.24 ± 6.49 | 0.0039 | — |
| Sex | 1.0: 54 (59.3%); 0.0: 37 (40.7%) | 1.0: 28 (60.9%); 0.0: 18 (39.1%) | 1.0: 26 (57.8%); 0.0: 19 (42.2%) | 0.8325 | — |
| Education years | 13.56 ± 3.55 | 14.71 ± 2.89 | 12.61 ± 3.79 | 0.0082 | — |
| Disease duration | 8.36 ± 4.24 | 8.04 ± 3.18 | 8.68 ± 5.11 | 0.4808 | — |
| UPDRS-III (Off) | 39.28 ± 16.56 | 36.86 ± 15.14 | 41.46 ± 17.64 | 0.2194 | 0.515 |
| MoCA | 24.43 ± 4.10 | 27.56 ± 1.61 | 21.30 ± 3.38 | <0.001 | <0.001 |
| BMI | 24.01 ± 2.99 | 23.71 ± 2.68 | 24.30 ± 3.28 | 0.3629 | 0.1576 |
| Triglycerides | 1.23 ± 0.93 | 1.11 ± 0.74 | 1.08 ± 0.77 | 0.5107 | 0.6786 |
| Uric acid | 308.79 ± 76.05 | 311.34 ± 62.66 | 306.31 ± 87.91 | 0.7698 | 0.9669 |
| MPI | 0.44 ± 0.20 | 0.46 ± 0.19 | 0.42 ± 0.21 | 0.4405 | 0.7682 |

**Table S3.** Clinical characteristics at the end of follow-up (T1) comparing converters and stable individuals.

Continuous variables are presented as mean ± standard deviation (SD), and categorical variables are presented as counts (percentages). P values were calculated using appropriate statistical tests. Age-adjusted P values were obtained using regression models with baseline age as a covariate. T1 represents the time point at the end of follow-up or the visit at which cognitive status was assessed.

**Figure S1.** Component-specific sensitivity analyses of TabPFN performance for first-recorded PD-MCI and PDD.

Panel A shows the discrimination of NCI versus first-recorded PD-MCI, and Panel B shows NCI versus first-recorded PDD. ROC curves were constructed from pooled out-of-fold predictions obtained using five-fold patient-level cross-validation with the same 10 prespecified predictors as the primary analysis. Shaded confusion matrices were calculated using the Youden thresholds derived from the corresponding out-of-fold predictions. AUC confidence intervals were estimated using 5,000 patient-level bootstrap resamples. The PDD-specific analysis should be interpreted as exploratory because it included only 15 PDD events.

**Figure S2.** Internal validation of the MoCA-only TabPFN model

(A) Receiver operating characteristic curve for the TabPFN model using baseline Montreal Cognitive Assessment (MoCA) score as the sole predictor. The model achieved an AUC of 0.742 (95% CI 0.599–0.826) under patient-level stratified five-fold cross-validation. (B) Confusion matrix based on the model-specific optimal threshold of 0.680, selected using the Youden index. The corresponding sensitivity and specificity were 44.4% and 93.5%, respectively. CI, cognitive impairment; NCI, no cognitive impairment.

**Figure S3.** Internal validation of the MoCA-excluded TabPFN model

(A) Receiver operating characteristic curve for the TabPFN model after excluding the Montreal Cognitive Assessment (MoCA) score while retaining the other nine predictors from the primary model. The model achieved an AUC of 0.693 (95% CI 0.558–0.782) under patient-level stratified five-fold cross-validation. (B) Confusion matrix based on the model-specific optimal threshold of 0.443, selected using the Youden index. The corresponding sensitivity and specificity were 80.0% and 52.2%, respectively. CI, cognitive impairment; NCI, no cognitive impairment.

**Figure S4.** External validation of the TG-excluded TabPFN model in the PPMI cohort.

The TabPFN model was rerun after triglycerides (TG) were excluded from both the internal training context and the PPMI external-validation dataset, while the remaining nine predictors and modeling procedures were unchanged. (A) Receiver operating characteristic curve for predicting cognitive impairment (CI) in the PPMI cohort. The model achieved an AUC of 0.740 (95% CI, 0.695–0.783). (B) Confusion matrix, the model achieved a sensitivity of 75.0% and a specificity of 64.4%. NCI, no cognitive impairment; CI, cognitive impairment; PPMI, Parkinson’s Progression Markers Initiative; TG, triglycerides.

**Figure S5.** Calibration curve of the prediction model in the internal validation cohort.

Calibration of the prediction model was assessed using a calibration curve with predicted probabilities grouped into quantile-based bins. The dashed diagonal line represents perfect calibration. The solid line represents the observed calibration performance of the model. The Brier score is reported as a measure of overall calibration accuracy. Each point corresponds to the mean predicted probability and the observed event rate within each bin.

| Variable | β | 95% CI lower | 95% CI upper | P value |
| --- | --- | --- | --- | --- |
| Intercept | -0.010 | -0.274 | 0.254 | 0.941 |
| Time (years) | 0.065 | -0.005 | 0.134 | 0.068 |
| Converter group | 0.266 | -0.156 | 0.687 | 0.217 |
| Time × Converter interaction | -0.154 | -0.278 | -0.030 | 0.015 |
| Baseline age | -0.003 | -0.020 | 0.014 | 0.740 |
| Sex | -0.098 | -0.432 | 0.235 | 0.563 |
| Diabetes | -0.107 | -0.522 | 0.308 | 0.614 |
| Hypertension | 0.069 | -0.301 | 0.439 | 0.714 |
| Baseline BMI | 0.034 | -0.020 | 0.088 | 0.218 |

**Table S4.** Pre-conversion sensitivity analysis of longitudinal log-transformed TG

Values are β coefficients, 95% confidence intervals, and two-sided P values from a random-intercept linear mixed-effects model. The analysis included 42 patients and 90 TG measurements: 32 Stable patients (68 measurements) and 10 Converters (22 measurements). Only cognitively unimpaired visits before the first documented cognitive-impairment visit were included, and patients were required to have at least two eligible TG measurements. The model was adjusted for baseline age, sex, diabetes, hypertension, and BMI. The Stable group was the reference. The Converter-group time slope was calculated as the sum of the time and time-by-Converter coefficients. β coefficients are expressed on the log(TG) scale. Because only 10 Converters had sufficient pre-conversion measurements, this analysis should be considered exploratory. CI, confidence interval; NCI, no cognitive impairment; TG, triglycerides; BMI, body mass index.

| Metabolite | Class | VIP | FC | Log2FC | P_value | NegLog10P | FDR | Significance |
| --- | --- | --- | --- | --- | --- | --- | --- | --- |
| Cer(d41:2)-Cer(d18:2/23:0) | Ceramide | 1.196812747 | 0.496677937 | -1.009617434 | 0.002026511 | 2.693251034 | 0.036443827 | Down-regulated |
| Cer(d41:1)-Cer(d18:1/23:0) | Ceramide | 1.288748676 | 0.592604315 | -0.754858964 | 0.000281638 | 3.550308549 | 0.017425806 | Down-regulated |
| Cer(d42:3)-Cer(d18:2/24:1) | Ceramide | 1.717708931 | 0.528047421 | -0.921260599 | 0.00027365 | 3.562804977 | 0.017425806 | Down-regulated |
| Cer(d42:2)-Cer(d18:1/24:1) | Ceramide | 1.737238245 | 0.558312513 | -0.840855205 | 0.000171019 | 3.766955583 | 0.01619771 | Down-regulated |
| Cer(d43:1)-Cer(d18:1/25:0) | Ceramide | 1.425937294 | 0.649288301 | -0.623068881 | 0.001475843 | 2.830959783 | 0.034721153 | Down-regulated |
| ChE(20:5)-ChE(20:5) | Cholesterol ester | 2.60050094 | 0.330144177 | -1.598831893 | 0.000311871 | 3.506024661 | 0.017425806 | Down-regulated |
| ChE(22:6)-ChE(22:6) | Cholesterol ester | 2.108048898 | 0.51197585 | -0.965852336 | 0.001402634 | 2.8530556 | 0.034721153 | Down-regulated |
| PC(39:6)-PC(39:6) | Glycerophosphocholine | 2.818130059 | 0.425812153 | -1.23171097 | 7.91E-05 | 4.102076353 | 0.01619771 | Down-regulated |
| PC(36:5p)-PC(36:5p) | Glycerophosphocholine | 2.487633444 | 0.407908086 | -1.29368399 | 0.001364561 | 2.865007186 | 0.034721153 | Down-regulated |
| PC(36:5e)-PC(16:0e/20:5) | Glycerophosphocholine | 2.130764559 | 0.444446584 | -1.169918056 | 0.002195865 | 2.658394442 | 0.0370007 | Down-regulated |
| PC(36:6)-PC(36:6)1 | Glycerophosphocholine | 1.326924415 | 0.403387203 | -1.309762778 | 0.001864513 | 2.72943468 | 0.036443827 | Down-regulated |
| PC(36:5)-PC(16:0/20:5) | Glycerophosphocholine | 1.933114838 | 0.50868721 | -0.975149275 | 0.001341802 | 2.872311603 | 0.034721153 | Down-regulated |
| PC(39:6)-PC(17:0/22:6) | Glycerophosphocholine | 1.792990917 | 0.658076353 | -0.603673112 | 0.001168988 | 2.9321899 | 0.034721153 | Down-regulated |
| PC(39:5)-PC(39:5) | Glycerophosphocholine | 1.561789874 | 0.650162231 | -0.621128346 | 0.001904219 | 2.720283198 | 0.036443827 | Down-regulated |
| PE(38:6p)-PE(16:0p/22:6) | Glycerophosphoethanolamine | 1.580163701 | 0.623199526 | -0.682233959 | 0.002038245 | 2.690743538 | 0.036443827 | Down-regulated |
| PE(40:7p)-PE(18:1p/22:6) | Glycerophosphoethanolamine | 1.648632283 | 0.568298434 | -0.815279356 | 0.000986028 | 3.006110637 | 0.034721153 | Down-regulated |
| PE(40:6p)-PE(18:0p/22:6) | Glycerophosphoethanolamine | 1.783272975 | 0.57125187 | -0.807801111 | 0.004220014 | 2.37468611 | 0.048367853 | Down-regulated |
| LysoPC(20:5)-LysoPC(0:0/20:5) | LysoPC | 2.046068685 | 0.458392621 | -1.125344273 | 0.00042466 | 3.3719589 | 0.021091434 | Down-regulated |
| SM(d37:1)-SM(d37:1) | Sphingomyelin | 1.809818661 | 0.559616667 | -0.837489163 | 1.39E-05 | 4.857709935 | 0.00620294 | Down-regulated |
| SM(d40:3)-SM(d20:0/20:3) | Sphingomyelin | 1.615238057 | 0.635804308 | -0.653345302 | 0.000864214 | 3.063378788 | 0.034721153 | Down-regulated |
| SM(d40:2)-SM(d22:0/18:2)1 | Sphingomyelin | 1.544539767 | 0.609217282 | -0.714971226 | 0.002359169 | 2.627240933 | 0.037662449 | Down-regulated |
| SM(d41:2)-SM(d41:2) | Sphingomyelin | 1.68777803 | 0.647572536 | -0.626886294 | 0.001873392 | 2.727371259 | 0.036443827 | Down-regulated |
| TG(62:14)-TG(18:2/22:6/22:6) | Triacylglycerol | 3.006206401 | 0.303319529 | -1.721089706 | 0.002705318 | 2.567781601 | 0.03983944 | Down-regulated |
| TG(62:13)-TG(18:1/22:6/22:6) | Triacylglycerol | 3.023299201 | 0.278898473 | -1.842188061 | 0.002984871 | 2.525074464 | 0.03983944 | Down-regulated |
| TG(60:13)-TG(20:5/18:2/22:6) | Triacylglycerol | 3.055599124 | 0.321866171 | -1.635467141 | 0.001320141 | 2.879379534 | 0.034721153 | Down-regulated |
| TG(60:12)-TG(16:0/22:6/22:6) | Triacylglycerol | 2.756683444 | 0.32891451 | -1.604215439 | 0.003088075 | 2.510312203 | 0.03983944 | Down-regulated |
| TG(54:5+O)-TG(18:1+O/18:2/18:2) | Triacylglycerol | 1.271476235 | 3.752967211 | 1.908031686 | 0.001585299 | 2.799888841 | 0.03543143 | Up-regulated |

**Table S5.** Differentially expressed lipid species between CI and NCI groups identified by lipidomic analysis.
